# Main-duct intraductal papillary mucinous adenoma of the pancreas

**DOI:** 10.1186/1477-7819-9-153

**Published:** 2011-11-23

**Authors:** Kensuke Takuma, Terumi Kamisawa, Taku Tabata, Masanao Kurata, Goro Honda, Shin-ichiro Horiguchi

**Affiliations:** 1 Departments of Internal Medicine, Tokyo Metropolitan Komagome Hospital, Tokyo, Japan; 2 Departments of Surgery, Tokyo Metropolitan Komagome Hospital, Tokyo, Japan; 3 Pathology, Tokyo Metropolitan Komagome Hospital, Tokyo, Japan

**Keywords:** intraductal papillary mucinous neoplasm, main duct, mural nodule, IPMN

## Abstract

**Background:**

The prevalence of carcinoma in main-duct intraductal papillary mucinous neoplasm (IPMN) is high, and surgical resection is recommended for all patients with a main-duct IPMN.

**Results:**

A main-duct IPMN with typical imagings including protruding lesions in the dilated main pancreatic duct was resected, but the histology was intraductal papillary mucinous adenoma of the pancreas.

**Discussion:**

It has been reported that the presence of mural nodules and dilatation of MPD are significantly higher in malignant IPMNs. The presented case had protruding lesions in the dilated main pancreatic duct on endoscopic ultrasonography, but the histology was adenoma.

**Conclusion:**

Preoperative distinction between benign and malignant IPMNs is difficult.

## Background

Intraductal papillary mucinous neoplasm (IPMN) of the pancreas is a distinct entity characterized by intraductal papillary growth and thick mucus secretion. Copious mucous fills the main and branch pancreatic ducts and causes ductal dilation. Thus, IPMNs of the pancreas are being diagnosed with increasing frequency by radiologic findings.

IPMNs are pathologically classified into two types based on site of tumor involvement, that is, main-duct type or branch type. The histology is classified into intraductal adenocarcinoma, intraductal adenoma, and hyperplasia. It has been suggested that these lesions have the capacity to progress from hyperplasia to adenoma, to noninvasive carcinoma, and ultimately to invasive carcinoma. The prevalence of carcinoma in main-duct IPMN is as high as 60% [1] to 92% [2], and international consensus guidelines [3] recommend surgical resection for all patients with a main-duct IPMN. In preoperative diagnosis, it is evaluated the existence of mural nodule increase the risk of malignancy [3-5]. Whereas, there are various unanswered problems about the conditions of main-duct type IPMNs. It is unknown whether all IPMNs have malignant potential or what the time course of progression may be.

## Case presentation

**Figure 1:**
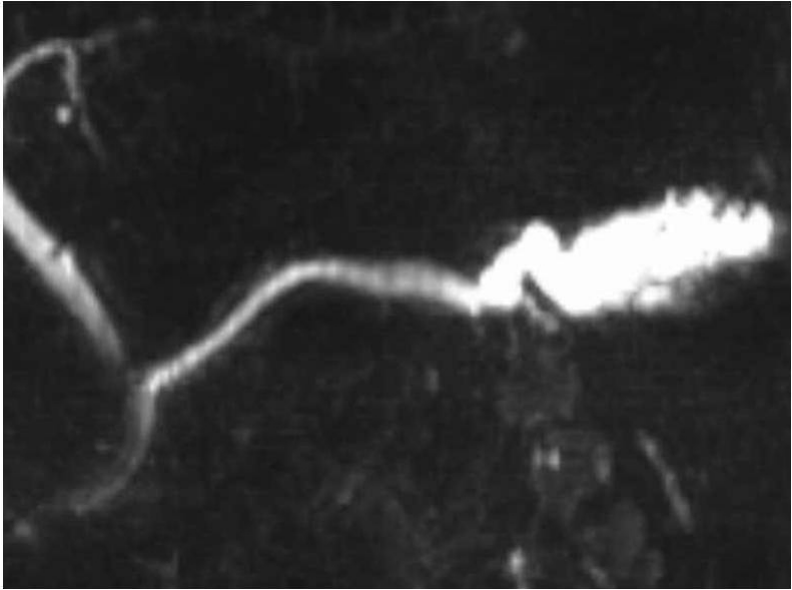
**MRCP of the case**. MRCP showing an irregular dilatation (10 mm) of the main pancreatic duct of the tail.

**Figure 2:**
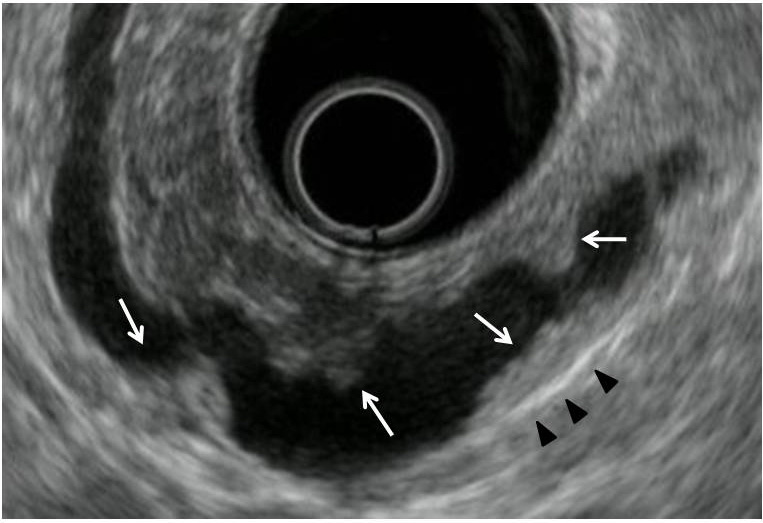
**EUS of the case**. EUS showing hyperplastic or a polypoid growth of the epithelial layer (arrows) and hyperechoic ductal margin (arrow heads) of the irregularly dilated main pancreatic duct.

**Figure 3:**
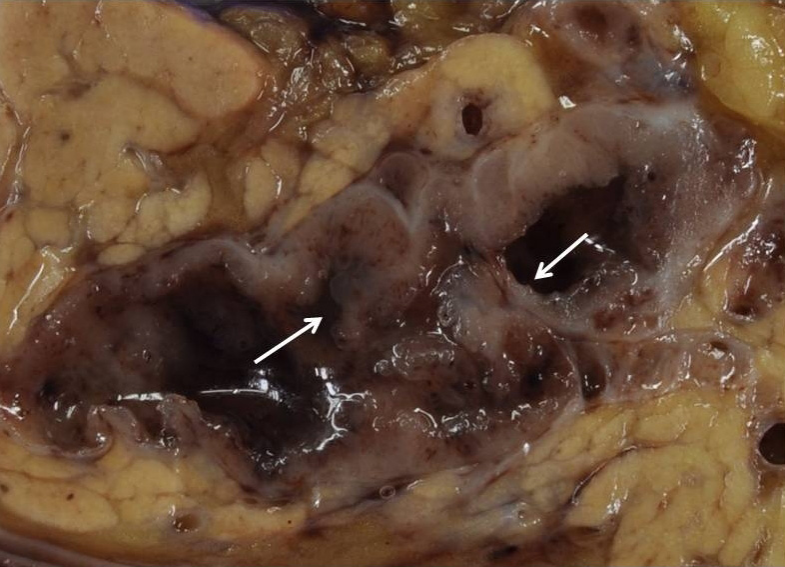
**Cut surface of the resected specimen**. Cut surface of the resected specimen showing hyperplastic or polypoid lesions (arrows) developed in the markedly thickened wall of the main pancreatic duct.

**Figure 4:**
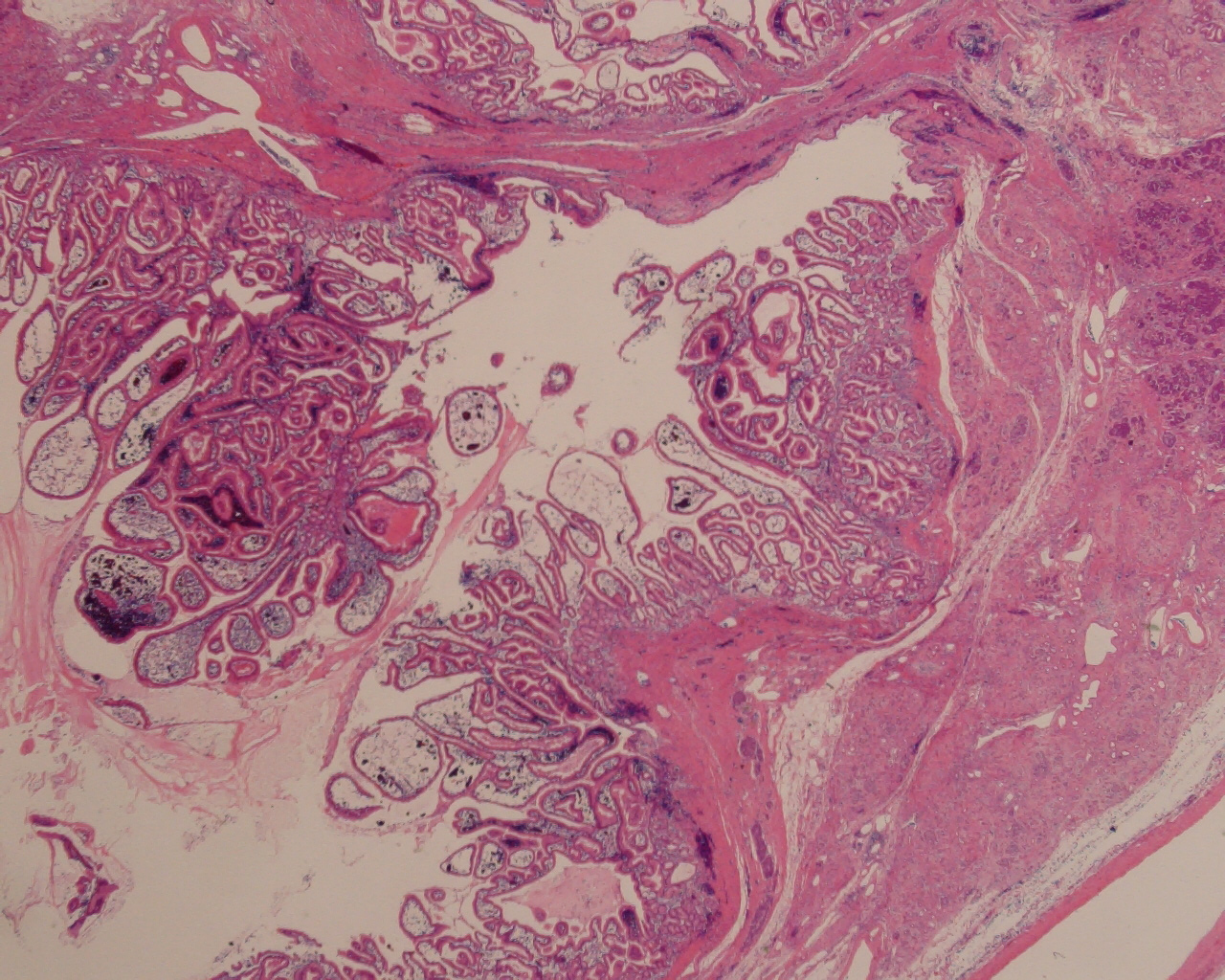
**Histology of the tumor**. Histologically, the tumor was classified as an intraductal papillary mucinous adenoma extensively proliferating in the main pancreatic duct. Fibrotic lesions with acinar atrophy surrounded the main pancreatic duct.

A 56-year-old man presented with mild abdominal pain. Physical examination and laboratory data were unremarkable. Abdominal symptoms were relieved with conservative medical management within a few days. Abdominal ultrasonography showed dilation of the main pancreatic duct (MPD). Subsequent computed tomography scan showed dilatation of the MPD and several small cysts in the pancreatic tail, with no findings of mural nodules, mass formation, metastasis, or lymph node swelling. Magnetic resonance cholangiopancreatography (MRCP) showed an irregular dilatation (10 mm) of the MPD of the tail [Figure 1]. On duodenoscopy, mucin was secreted from a patulous orifice of the papilla of Vater. On endoscopic retrograde pancreatography, a filling defect equivalent to mucus was shown in the dilated MPD. Cytological examination of the pancreatic fluid was negative, and CEA level in the pancreatic fluid was 2.3 ng/ml. Endoscopic ultrasonography (EUS) revealed hyperplastic or polypoid growth of the epithelial layer and hyperechoic ductal margin of the irregularly dilated MPD [Figure 2]. Under a diagnosis of main-duct IPMN, laparoscopy-assisted distal pancreatectomy was performed. Cut surface of the resected specimen revealed hyperplastic or polypoid lesions developed in the markedly thickened wall of the MDP [Figure 3]. Histologically, the tumor was classified as intraductal papillary mucinous adenoma extensively proliferating in the MPD, and the surgical margin was free. Immunohistochemically, Ki-67 labeling index was 1.8% and p53-positive cells were not observed in the tumor cells. Fibrotic lesions with acinar atrophy surrounded the MPD [Figure 4].

## Discussion

IPMN is an increasingly recognized entity representing a spectrum of benign and malignant neoplasms of the pancreas. International consensus guidelines [3] recommend surgical resection for all patients with a main-duct IPMN because of high potential for complication of malignancy.

It has been reported that the presence of mural nodules and dilatation of MPD are significantly higher in malignant IPMNs [3]. We also reported MPD diameter ≥ 10 mm and appearance of mural nodules were significantly more frequent in malignant IPMNs [4]. Sugiyama et al. reported that presence of symptoms, MPD diameter > 15 mm, and mural nodules were significant predictors of malignancy in main-duct or mixed type IPMNs [5]. In Sang et al. report, multivariate analysis showed that age more than 60 years, history of pancreatitis, presence of mural nodules, MPD dilatation > 6 mm were independent predictors of invasive IPMNs [6]. However, there were some typical main-duct IPMN cases that pathologically confirmed to be benign, and could be followed-up for long term [3-5,7].

EUS is one of the most useful modalities to evaluation local changes in the pancreas. Intraductal papillary adenocarcinoma is characterized by papillary protrusions and thick septum like structures in a dilated duct as delineated by EUS. When the thickness of the septum structure is more than 4 mm on EUS, neoplastic change should be suspected. Finding of solid mass or a mass showing a mixed-echo pattern in the pancreatic parenchyma are characteristic of the invasive type of IPMN [8].

The present case was a typical main-duct IPMN, and EUS showed its entire picture. Although the case had mural nodules and the dilated MPD which were indicators of possible malignancy, the histology was intraductal papillary mucinous adenoma. For the high prevalence of carcinoma in main-duct IPMNs, international consensus guidelines that recommend surgical resection for all patients with a main-duct IPMN are considered reasonable and proper. As it stands now, although the histology was adenoma, resection was considerable based on the patient' s age and adenoma-carcinoma sequence. Preoperative distinction between benign and malignant IPMNs remains difficult.

## Conclusion

Preoperative distinction between benign and malignant IPMNs remains difficult.

## Consent

Written informed consent was obtained from the patient for analysis of the resected specimen and imaging, and publication. A copy of the written consent is available for review by the Editor-in-Chief of this journal.

## Conflict of interest statement

The authors declare that they have no competing interests.

## Authors' contributions

KT, TK, and TT did the examination, KT and TK wrote the paper, MK and GH did the surgery, and SH did the pathology. All the authors reviewed and approved the end version.
